# Effects of Daratumumab Treatment on a Case of Overlapping Autoimmunity With Sjögren's Disease and Linear IgA Dermatosis

**DOI:** 10.1002/mco2.70881

**Published:** 2026-09-11

**Authors:** Carlijn Hut, Rob J. Volkering, Gwenny M. Verstappen, Jaap A. van Doesum, Frans G. M. Kroese, Barbara Horváth, Hendrika Bootsma, Tobit D. Steinmetz

**Affiliations:** ^1^ University Medical Center Groningen Groningen the Netherlands

1

Dear Editor:

Although sicca symptoms of the mouth and eyes are the most typical manifestation of Sjögren's disease (SjD), organ involvement is common, including skin, lung, kidney, musculoskeletal, and neurological complications. In rare cases, SjD may coincide with linear IgA dermatosis (LAD), an autoimmune blistering disease caused by IgA autoantibodies directed against the hemidesmosome protein BP180 [1]. Increased abundance of antibody‐secreting plasma cells is seen in the blood and salivary glands of SjD patients together with an altered phenotype, including increased maturity [2]. In LAD, IgA plasma cells accumulate at various mucosal sites. While the direct pathogenicity of autoantibodies in SjD is unclear, autoreactive IgA antibodies are known to be pathogenic in LAD. Plasma cell depletion can be achieved due to their high expression of CD38. Here, we evaluated the effects of the therapeutic anti‐CD38 antibody daratumumab in a patient with coincident SjD and LAD.

A 43‐year‐old woman was diagnosed with LAD and 3 years later with SjD (Figure 1A). By the time of inclusion into the REgistry of Sjögren Syndrome LongiTudinal cohort, she had minimal residual salivary flow rates of 0.0 mL/min unstimulated and 0.3 mL/min stimulated whole saliva, respectively. Titers for SSA/Ro52 and SSA/Ro60 autoantibodies were positive. On the skin, especially on the scalp and torso, she had multiple annular erythematous plaques with vesicles and crusts in the edges (string‐of‐pearls, Figure 1B). Routine direct immunofluorescence on perilesional skin showed IgA 3+ in a linear n‐serrated pattern along the epidermal basement membrane zone. On histopathologic evaluation, subepidermal blister formation was seen with predominantly neutrophilic granulocytes and only a few eosinophilic granulocytes. Prior treatments of the patient primarily targeting the LAD included adalimumab, rituximab, prednisolone, colchicine, MMF, methotrexate, cyclosporine, cyclophosphamide, leflunomide, and hydroxychloroquine. Nevertheless, the patient reported consistently high SjD‐related symptom burden assessed with the European Sjögren Syndrome Patient Reported Index (ESSPRI).

**FIGURE 1:**
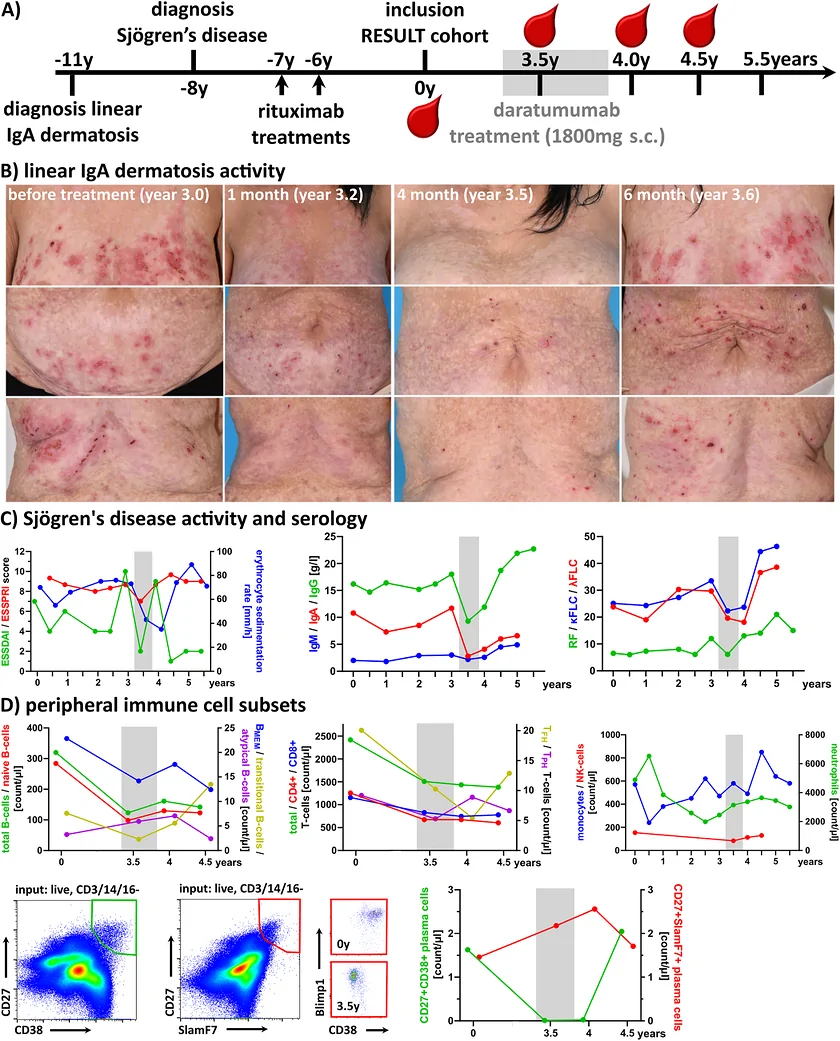
Changes in disease characteristics during the treatment timeline. (A) Case timeline indicating blood sampling and daratumumab administration timepoints, changes over time in (B) Linear IgA dermatosis (LAD) activity on the chest (upper panel), abdomen (middle panel), and back (lower panel), and (C) Sjögren's disease (SjD) activity and patient serology. (D) Changes in the absolute immune cell numbers in counts/µl blood for major B‐cell, major T‐cell, major innate immune cell, and plasma cell populations, including conventional (green) and alternative (red) gating of plasma cells. Peripheral blood mononuclear cell (PBMC) samples taken at 0, 3.5, 4, and 4.5y timepoints; y = year (relative to RESULT inclusion); s.c. = subcutaneous; RF = rheumatoid factor; κ/λFLC = kappa / lambda free light chain; grey area = daratumumab treatment interval.

Plasma cell‐targeting was chosen as a new experimental therapy option for this patient with overlapping SjD and LAD. Therefore, 1800 mg daratumumab was subcutaneously administered to the patient weekly for 4 weeks, followed by a biweekly scheme for 5 months. Mild side effects, including headache, eye complaints, and nausea, occurred.

Daratumumab treatment was initiated at the 3.15‐year timepoint (3 years and 2 months) of the cohort timeline. Within 1 month, the skin was in almost complete remission. At the 3.5‐year routine visit, total European Sjögren Syndrome Disease Activity Index (ESSDAI), total ESSPRI, and ESR decreased (Figure 1C). However, the dryness domain of the ESSPRI and the biological domain of the ESSDAI remained unaffected, the latter due to continuous persistence of cryoglobulins. All measured plasma cell‐related secretion products decreased during daratumumab treatment (Figure 1C), except anti‐nuclear, anti‐SSA, or anti‐SSB antibodies (data not shown). With the context of recurrent skin infections, the patient had a flare with *Streptococcus agalactiae* (group B) and herpes simplex virus type 1 coinfection of the skin, successfully treated with clindamycin and valaciclovir within 2–3 months. Later, another secondary infection with *Staphylococcus aureus* and *Streptococcus dysgalactiae*/*canis* occurred with no effect of antibiotic treatment but self‐resolving over time. Skin infections during daratumumab treatment are known from clinical trials, e.g., in light‐chain amyloidosis [3], but might be more likely with pre‐existing skin lesions or immunosuppressive medication in this patient. Three months after discontinuation of daratumumab injections (4‐year timepoint), ESSDAI, ESSPRI, and rheumatoid factor increased again to levels comparable to the pre‐treatment timepoint. ESR, IgA, IgG, and free light chains remained unchanged 3 months after discontinuation, but increased to previous levels during follow‐up.

Longitudinal blood lymphocyte analysis using flow cytometry (Supporting Information) revealed a decrease in total B‐cell numbers upon daratumumab treatment. CD11c+Tbet+ atypical B‐cells temporarily increased in numbers (Figure 1D), possibly driven by less competition for B‐cell survival cytokines. Total T‐cells, but also CD4+, CD8+, and follicular and peripheral T‐cell helper populations, declined during the observation period (Figure 1D). Conventional flow cytometry detection indicated a complete absence of plasma cells, defined as CD27+CD38+ cells (Figure 1D), due to daratumumab. However, assessment with the alternative pan‐plasma cell marker SlamF7 [4] revealed that plasma cells, defined as CD27+SlamF7+, were continuously present in the blood (Figure 1D). Blimp1 expression, the major plasma cell transcription factor, confirmed the plasma cell identity of the CD27+SlamF7+ cells with undetectable CD38 expression.

While no reports of daratumumab use in LAD are published, our observations of reduced general antibody titers and declined disease activity are comparable to a similar report in SjD [5]. The effects of daratumumab seemed to be transient, as the patient‐reported symptoms and serological parameters reversed upon follow‐up after discontinuation of daratumumab with hydroxychloroquine as maintenance therapy. ESSDAI scores remained low during follow‐up, with the exception of a short “relapse” directly after daratumumab discontinuation, due to a transient increase in the constitutional and lymphadenopathy domains.

In‐depth lymphocyte assessment revealed a marked reduction of B‐cells, mainly affecting naïve and transitional B‐cells. Since transitional B‐cells, either defined as CD24+CD38+ B‐cells or alternatively as CD10+CD24+ B‐cells, were reduced, fewer circulating naïve B‐cells are likely the result of reduced input from early B‐cell differentiation stages. Circumventing CD38‐masking by detecting plasma cells with SlamF7 revealed that daratumumab failed to deplete plasma cells from the circulation, underlining that SlamF7 detection on plasma cells in the context of daratumumab is crucial. The distinct reduction of plasma cell products (antibodies and free light chains) suggests systemic depletion of plasma cells in the tissues. The turnover of plasma cells entering and leaving the circulation might be undisturbed. Daratumumab‐labeled circulating plasma cells might be poised to die, as indicated by reduced expression levels of anti‐apoptotic Mcl1 and the pro‐survival receptor CD28 (data not shown). Insufficient depletion by phagocytes or complement, but inhibition of cell adhesion or enzymatic functions of CD38 could alternatively explain reduced plasma cell output with a persistent high plasma cell number in the blood. Reduced plasma cell expression of CD19 and CD20 (data not shown), coinciding with daratumumab treatment, suggests a higher susceptibility of immature plasma cells. Since we previously linked increased plasma cell maturity to more severe SjD features [2], persistent amelioration of SjD symptoms might require longer daratumumab treatment to deplete long‐lived plasma cells with concomitant effects on autoantibody levels.

## Author Contributions

**C. H**.: formal analysis; investigation; visualization; writing – review & editing. **R. J. V**.: data curation; formal analysis; investigation; writing – review & editing. **G. M. V**.: conceptualization; funding acquisition; supervision; writing – review & editing. **J. A. van D**.: data curation; formal analysis; investigation; writing – review & editing. **F. G. M. K**.: conceptualization; supervision; validation; writing – review & editing. **B. H**.: conceptualization; data curation; formal analysis; investigation; supervision; validation; visualization; writing – review & editing. **H. B**.: conceptualization; data curation; supervision (lead); validation; writing – review & editing. **T. D. S**.: conceptualization (lead); data curation; formal analysis; funding acquisition; investigation; supervision; validation; visualization; writing – original draft preparation; writing – review & editing (lead).

Dr. Steinmetz had full access to all of the data in the study and takes responsibility for the integrity of the data and the accuracy of the data analysis. All authors were involved in drafting the article or revising it critically for important intellectual content, and all authors have read and approved the final manuscript.

## Funding

This work is funded by a grant from the German Research Foundation (Deutsche Forschungsgemeinschaft, DFG)—project number 456067883 to Tobit D. Steinmetz. This publication is part of project numbers 09150162010166 and 09150162210226 of the Veni research program to Gwenny M. Verstappen and Tobit D. Steinmetz, respectively, which are financed by the Dutch Research Council (NWO).

## Ethics Statement

This study is part of the Registry of Sjögren's syndrome in University Medical Center Groningen Longitudinal (RESULT) cohort with ethics approval from the University Medical Center Groningen institutional review board (approval number METc 2014/491).

## Consent

The described participant provided an additional written consent for this study and the content of this publication.

## Conflicts of Interest

The authors declare no conflicts of interest.

## Supporting information




**Supporting Information File 1**: mco270881‐Supp‐0001‐SuppMatt.docx

## Data Availability

The data used and analyzed in this study are available from the corresponding author on reasonable request.
