# Supplementary material for: Effects of Daratumumab Treatment on a Case of Overlapping Autoimmunity With Sjögren's Disease and Linear IgA Dermatosis
Source: MedComm (2020). 2026 Sep 11;7(9):e70881. doi: 10.1002/mco2.70881 (PMC13563381; doi:10.1002/mco2.70881)
Supplement: Supplementary file 1 [file MCO2-7-e70881-s001.docx]

**Supplementary Materials for**

**Effects of daratumumab treatment on a case of overlapping autoimmunity with Sjögren’s disease and linear IgA dermatosis**

Carlijn Hut^1^, Rob J. Volkering MD^1^, Gwenny M. Verstappen PhD PharmD^1^, Jaap A. van Doesum MD^1^, Frans G.M. Kroese PhD^1^, Barbara Horváth MD PhD^1^, Hendrika Bootsma MD PhD^1^, Tobit D. Steinmetz PhD^1^

^1^University Medical Center Groningen, Groningen, The Netherlands

***Corresponding author:**

Tobit D. Steinmetz, PhD

E-mail: [d.t.steinmetz@umcg.nl](mailto:d.t.steinmetz@umcg.nl)

Tel: +31-50-3616601

Department of Rheumatology and Clinical Immunology

University Medical Center Groningen

Hanzeplein 1, PO Box 30.001, 9700RB Groningen, The Netherlands

**Materials and methods**

**Flow cytometry staining of frozen peripheral blood mononuclear cells**

Flow cytometry staining and data processing was performed as described previously² (Steinmetz et al. 2022) using frozen peripheral blood mononuclear cells. In brief, thawed cells were incubated in 1:20 TruStain Fc receptor-blocking reagent (BioLegend, Cat# 422302) and 1:200 Zombie green viability staining (BioLegend, Cat# 423111) for 10 minutes at room temperature (RT). Cells were washed in 1 ml PBS, 0.5% BSA, 2mM EDTA and resuspended in the surface staining cocktail containing the following antibodies: CXCR3-BUV395 (BD Biosciences, Cat# 565223), CD45RA-BUV563 (BD Biosciences, Cat# 612927), SlamF7-BUV661 (BD Biosciences, Cat# 750834), CD10-BUV737 (BD Biosciences, Cat# 612826), CD21-BV421 (BD Biosciences, Cat# 562966), CD19-eF450 (BioLegend, Cat# 512312), CD49d-BV510 (BioLegend, Cat# 304318), CD44-BV570 (BioLegend, Cat# 103037), CD11c-BV605 (BioLegend, Cat# 350522), CD27-BV650 (BioLegend, Cat# 302828), CD86-BV711 (BioLegend, Cat# 305439), PD-1-BV786 (BD Biosciences, Cat# 563789), CXCR5-BB515 (BD Biosciences, Cat# 564624), CD14-FITC (BD Biosciences, Cat# 345784), CD16-FITC (BD Biosciences, Cat# 335035), CD11a-PerCP (BioLegend, Cat# 350608), CD8-PerCP-Cy5.5 (BD Biosciences, Cat# 565310), CD24-PE-D594 (BioLegend, Cat# 311910), CD138-PE-Cy5 (BioLegend, Cat# 356548), CD56-PE-Fire700 (BioLegend, Cat# 392428), CD28-PE-Cy7 (BioLegend, Cat# 302926), CD20-AF700 (BioLegend, Cat# 302322), CD3-AF700 (Invitrogen, Cat# 56-0038-42), CD38-APC-eF780 (Invitrogen, Cat# 47-0389-42) and HLA-DR-PE-Fire810 (BioLegend, Cat# 307683). Cells were incubated for 30 minutes at RT in the dark and then washed in 1 ml PBS, 0.5% BSA, 2mM EDTA. Cell fixation was performed using the FoxP3 intracellular staining kit (eBioscience, Cat# 00-5523-00) according to manufacturer’s instructions incubating the cells for 30 minutes at RT in the dark. Two washing steps with 1 ml PBS, 0.5% BSA, 2mM EDTA and 1 ml permeabilization buffer (see FoxP3 kit) were performed, followed by resuspending the cells in the intracellular staining cocktail with the following antibodies: IgG-BV480 (BD Biosciences, Cat# 746341), IgM-PerCP-Cy5.5 (BioLegend, Cat# 314512), Blimp1-PE (BD Biosciences, Cat# 564702), Mcl1-APC (Invitrogen, Cat# 17-9047-42) and T-Bet-eF660 (Invitrogen, Cat# 50-5825-82). Cells were incubated for 30 minutes at RT in the dark and then washed in 1 ml permeabilization buffer and then 1 ml PBS, 0.5% BSA, 2mM EDTA. Finally, resuspended cell suspensions were measured using the Cytek Aurora 5L spectral cytometer.
